# Acceptability and feasibility of mobile phone-based ecological momentary assessment and intervention in Uganda: A pilot randomized controlled trial

**DOI:** 10.1371/journal.pone.0273228

**Published:** 2022-08-26

**Authors:** Laura K. Beres, Ismail Mbabali, Aggrey Anok, Charles Katabalwa, Jeremiah Mulamba, Alvin G. Thomas, Eva Bugos, Mary K. Grabowski, Gertrude Nakigozi, Larry Chang

**Affiliations:** 1 Department of International Health, Johns Hopkins Bloomberg School of Public Health, Baltimore, MD, United States of America; 2 Rakai Health Sciences Program, Entebbe, Uganda; 3 Department of Epidemiology, University of North Carolina, Chapel Hill, NC, United States of America; 4 Department of Surgery, Johns Hopkins University, Baltimore, MD, United States of America; 5 Department of Population, Family and Reproductive Health, Johns Hopkins Bloomberg School of Public Health, Baltimore, MD, United States of America; 6 Department of Medicine, University of Pittsburgh Medical Center, Pittsburgh, PA, United States of America; 7 Department of Pathology, Johns Hopkins University School of Medicine, Baltimore, MD, United States of America; 8 Department of Epidemiology, Johns Hopkins Bloomberg School of Public Health, Baltimore, MD, United States of America; 9 Division of Infectious Diseases, Department of Medicine, Johns Hopkins University School of Medicine, Baltimore, MD, United States of America; PLOS: Public Library of Science, UNITED KINGDOM

## Abstract

Valid, reliable behavioral data and contextually meaningful interventions are necessary for improved health outcomes. Ecological Momentary Assessment and Intervention (EMAI), which collects data as behaviors occur to deliver real-time interventions, may be more accurate and reliable than retrospective methods. The rapid expansion of mobile technologies in low-and-middle-income countries allows for unprecedented remote data collection and intervention opportunities. However, no previous studies have trialed EMAI in sub-Saharan Africa. We assessed EMAI acceptability and feasibility, including participant retention and response rate, in a prospective, parallel group, randomized pilot trial in Rakai, Uganda comparing behavioral outcomes among adults submitting ecological momentary assessments (EMA) versus EMAI. After training, participants submitted EMA data on five nutrition and health risk behaviors over a 90-day period using a smartphone-based application utilizing prompt-based, participant-initiated, and geospatial coordinate data collection, with study coordinator support and incentives for >50% completion. Included behaviors and associated EMAI-arm intervention messages were selected to pilot a range of EMAI applications. Acceptability was measured on questionnaires. We estimated the association between high response rate and participant characteristics and conducted thematic analysis characterizing participant experiences. Study completion was 48/50 participants. Median prompt response rate was 66.5% (IQR: 60.0%-78.6%). Prior smartphone app use at baseline (aPR 3.76, 95%CI: 1.16–12.17, p = 0.03) and being in the intervention arm (aPR 2.55, 95% CI: 1.01–6.44, p = 0.05) were significantly associated with the top response rate quartile (response to >78.6% of prompts). All participants submitted self-initiated reports, covering all behaviors of interest, including potentially sensitive behaviors. Inconsistent phone charging was the most reported feasibility challenge. In this pilot, EMAI was acceptable and feasible. Response rates were good; additional strategies to improve compliance should be investigated. EMAI using mobile technologies may support improved behavioral data collection and intervention approaches in low and middle-income settings. This approach should be tested in larger studies.

## Introduction

Valid and reliable behavioral data and contextually meaningful interventions are necessary to support optimal health outcomes. Routine methods for assessing behaviors often involve periodic, retrospective self-report, which is de-contextualized, subject to recall bias and, particularly for routine behaviors, may have poor reliability [1]. Ecological Momentary Assessment (EMA) is a technique for gathering richer and more relevant data through repeated, context-sensitive, longitudinal sampling of participants [1]. Often leveraging mobile technologies, Ecological Momentary Assessment and Intervention (EMAI) collects data in a participant’s natural setting to deliver real-time or near real-time interventions [1]. Rapid emergence of mHealth (mobile technologies for health), including growing access to low-cost smartphones, now allows for unprecedented EMAI advancements in low and middle-income countries (LMICs) [2].

Several studies in high-income countries have shown EMA can be used to effectively collect behavioral data including dietary [3, 4], substance use [5–7], and sexual risk behaviors [8]. One small study among women in South Africa showed it feasible to collect daily sexual risk data over mobile phones [9]. EMA using mobile technology not only offers the opportunity for behavioral data collection, but can leverage geospatial data to contextualize behaviors and to develop effective interventions [10]. While there is less research on EMAI, several small studies have shown the feasibility of providing individualized feedback interventions through a smartphone application to support improved outcomes in patients with mental illness in high-resource settings [11, 12]. Both EMAI and the rapidly growing field of mHealth interventions require further research on acceptability and feasibility to optimize their use [13, 14]. Despite clear potential for improved data collection and intervention, no previous studies to our knowledge have collected geospatial information with EMA or trialed EMAI in sub-Saharan Africa. In the context of COVID-19, the importance of understanding and optimizing use of remote data collection and intervention approaches is more acute.

We previously reported on a prospective, parallel group, randomized pilot trial in Rakai, Uganda comparing behavioral outcomes among adults submitting ecological momentary assessments (EMA) versus EMAI, which offered preliminary evidence that EMAI may support behavior change in this setting [15]. To advance future implementation of EMAI and to better understand feasibility and acceptability, we now assess and report on implementation-related issues around using smartphones to collect near real-time, geolocated behavioral information and to send tailored health messages based upon these data. Including male and female adults of varying ages, we assessed both prompt-driven and participant-led event contingent self-report covering diet, alcohol, tobacco, and sexual behaviors. These behaviors were selected to pilot a range of possible EMAI applications, each with associated behaviorally-responsive intervention text-messages.

## Methods

### Study design and participants

The study was an assessment of the feasibility and acceptability of using mobile smartphones to implement EMAI in a prospective, parallel group, randomized pilot trial in Rakai, Uganda [15]. The trial purposively sampled adult participants (18–49 years) from the Rakai Community Cohort Study (RCCS), an open, population-based HIV and health surveillance cohort established in 1994 [16, 17]. The Rakai region, situated in south-central Uganda, is bordered by Tanzania and Lake Victoria, and includes agrarian, trading and fishing communities [17]. Eligible participants: 1) were current RCCS participants, 2) had at least a secondary-level education, and 3) had provided a telephone number in the last RCCS survey. Participants were purposively recruited via telephone from lists of potential participants who met eligibility criteria in the RCCS database. Study staff members sought variation in participant age, sex, and occupation, aiming to include a minimum of 20% traders and 20% farmers in the sample, to enable researchers to assess possible differential EMAI acceptability and feasibility by participant characteristics in this pilot trial. Due to unforeseen resource limitations, the study recruited approximately half the number expected in the study protocol. However, purposeful sampling ensured inclusion of the desired range of participant characteristics in the pilot trial [15]. Participants were informed of the study and, if they agreed to participate, were then contacted in-person and enrolled, contingent on their written voluntary informed consent. Rolling recruitment permitted up to ten simultaneous active participants. Participants were recruited between 15^th^ February 2016 – 1^st^ March 2017, with follow-up post enrolment through 90-days, ending on the 31^st^ May 2017.

The trial included a randomized component, with participants randomized to receiving health messaging or not at Day 30. Participants were assigned to the control or intervention study arm in a 1:1 ratio using block randomization with randomly varying block sizes of 4, 6, and 8 through blockr and R package by Greg Snow. Study arm assignments were enclosed within opaque, consecutively numbered envelopes, and allocated to participants consecutively at their 30-day visits by the study coordinator. Assignments were not masked to study participants or staff. This paper focuses on overall acceptability and feasibility of the pilot. Specifically, we report on feasibility outcomes including overall participant retention, proportion of EMA prompts to which participants responded (‘response rate’), event-contingent behavioral data collected, and participant-reported acceptability. We also examine associations between participant characteristics and response rate to inform future use of EMAI in LMICs.

The study was approved by the Ugandan Virus Research Institute Research and Ethics Committee and Johns Hopkins School of Medicine Institutional Review Boards. The trial was registered under NCT04375423 on ClinicalTrials.gov. Due to the pilot nature of the study, the trial was registered after participant enrolment began. The authors confirm that all ongoing and related trials for this intervention are registered.

#### Inclusivity in global research

Additional information regarding the ethical, cultural, and scientific considerations specific to inclusivity in global research is included in the S1 Checklist.

### Procedures

#### EMA reporting and messaging

Study participants submitted EMA data over a 90-day period using a smartphone-based application (app). Participants submitted three types of behavioral assessments: twice-daily, weekly, and event-contingent. Twice per day, once at a fixed time and once at a random time, participants received an audible alert and text-message prompt to complete a form recalling (i) fruit consumption, (ii) vegetable consumption, (iii) alcohol intake, (iv) cigarette smoking, (v) sex with a non-marital or non-long-term partner and condom use since the last form was completed. Weekly prompts requested completion of a cumulative seven-day form on the same behaviors. Participants were also requested to self-initiate and submit a report when they engaged in one of the five study behaviors of interest. Referred to as an ‘event-contingent report’, participants were instructed to submit within one hour of engaging in the behavior (i.e. self-initiating and submitting a form in the app whenever the participant smoked a cigarette, consumed fruit, vegetables or alcohol, or had sex with a non-marital or non-long-term partner) and the question structure asked about behavior in the hour prior to submission; however, actual time elapsed between the behavior and report was not possible to measure.

After an initial 30-day period of baseline data collection, participants were randomized 1:1 to health messaging. Those in the intervention arm began receiving behaviorally-responsive text messages. Based on information in submitted forms, the app delivered a message offering positive reinforcement or encouragement to change (e.g. ‘Great job going smoke-free today—keep up the good work’ or ‘Smoking can cause lung cancer. Make a decision to improve your health, and make tomorrow a smoke-free day’) to individuals in the intervention arm. Participants in the control arm continued to receive alerts and complete prompts as before.

**Fig 1:**
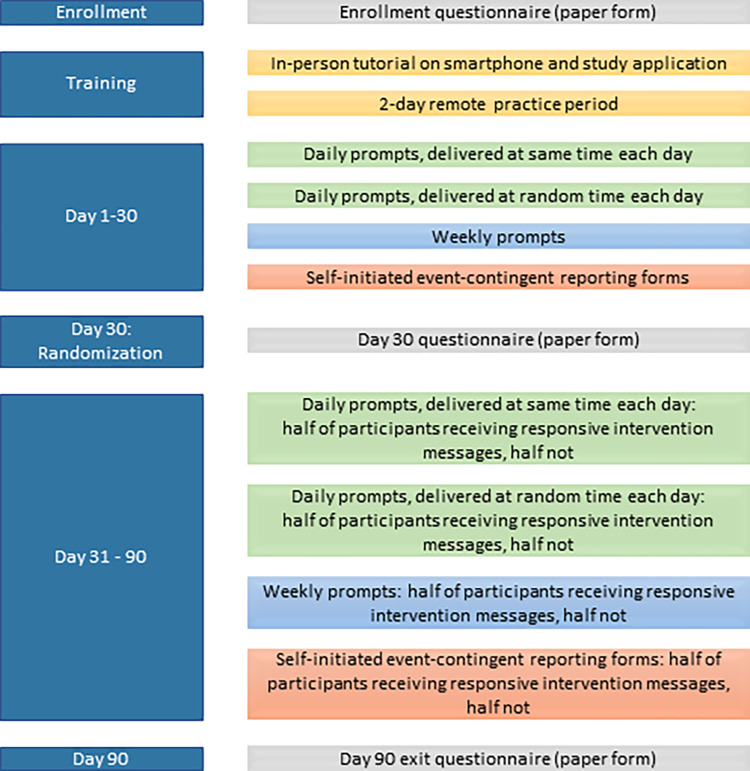
Study design. Figure partially reproduced from Beres et al, JMIR Form Res., 2021, https://doi.org/10.2196/22693.

Participants completed a paper-based questionnaire including demographics, phone usage and EMAI acceptability information at enrollment, 30, and 90-day in-person study visits. (Fig 1)

#### Equipment, software, and participant training

Enrolled participants were issued a password-protected 2015 Motorola Moto E smartphone (~80 USD) programmed with the EMAI study application (emocha Health Inc. Baltimore, MD), phone charger, and portable power bank. Phones were pre-programmed with study staff contact details in case outreach was needed. Phone data could be deleted remotely in event of loss or theft. Phones and equipment were returned at data collection completion.

All EMA prompts and completed forms were generated and stored on the phone, then sent to the remote study database for analysis in two separate data processing tasks. A unique identification number was created for each prompt generated, allowing the study database to identify the total number of prompts generated, even if prompt or response data were not received due to interrupted data transmission (e.g. battery charge or signal interruption). Twice-daily and weekly forms could only be sent in response to a generated prompt. If a prompt response was sent by the participant, the date and time when the associated response form was sent was also recorded in the database. Data generated from the mobile application were stored on a secure online server with authenticated access.

Participants engaged in a one-on-one, 1.5-hour training on smartphone and app use prior to study start. The first 48 hours post-training were a designated practice period. Study staff members monitored submissions and contacted the participant to assess if the practice period should be extended or if formal data collection could begin. At randomization, participants received refresher training, including information on intervention message receipt if in the intervention arm.

The Study Coordinator reviewed responses on the study data collection website every other day. Primarily in the first month of study participation, if participants did not respond to all sent prompts, the Study Coordinator would follow-up with a phone call or visit to encourage responses and navigate smartphone or app challenges.

Participants were given 20,000 Ugandan Shillings (UGX) monthly for data equivalent to 525MBs. All participants were compensated 10,000 UGX for their time (approximately 3 USD) and refunded travel expenses (5,000–40,000 UGX / 1.50–12 USD) for each study visit. Additionally, participants responding to ≥50% of prompts received a total of 100,000 UGX (approximately 30 USD) in three separate increments at 30, 60 and 90 days, with no incentives associated with event-contingent reports.

### Study measurements and outcomes

Participant demographics at enrollment, contact information, and smartphone usage experience were taken from the paper-based enrollment questionnaire. Whether or not a participant consumed fruits, vegetables or alcohol, smoked cigarettes, had sex with a non-marital or non-long-term partner, and used condoms with all such partners was taken from the daily, weekly and event-contingent app reporting forms (S1 Appendix). App forms recorded quantity for fruit and vegetable consumption and cigarette smoking. Weekly forms also recorded the number of days/week on which the behaviors occurred. Participant geospatial coordinates were automatically recorded by the app and sent to the study database both with form reports and every 4–6 hours. Participant challenges with the phone, EMAI likes and dislikes, and perceived study impact were recorded on the 30 and 90-day questionnaire in response to open-ended questions.

The number of submitted prompt response forms included all daily and weekly forms in the database with a time and date stamp recorded in the form itself, or in the associated prompt. If time and date were only recorded in the associated prompt, behavioral data were missing. The number of prompts sent was calculated as the total number of generated prompts counted in the database plus any submitted prompt response forms exceeding the number of counted generated prompts per participant, as a daily or weekly form could only be submitted in response to a prompt. Response forms exceeding the number of counted prompts indicate missing prompts. The number of submitted event-contingent reports was calculated as the total number of event-contingent submissions in the database per participant.

Outcomes included participant retention, defined as remaining in the study for the full 3 months of data collection; participant-related variation in response rate, defined as the proportion of prompts to which participants responded; availability of behavioral data from prompt-based and event-contingent EMA, availability of geospatial data, and participant experience measured through open-ended, short response questions upon study exit.

### Analysis

We used descriptive statistics to characterize participant demographics, behavioral data from EMA prompt-driven forms and event-contingent reports, and geospatial data. Reports on quantity of fruits, vegetables and cigarettes consumed were examined for implausibly large values using box plots, excluding outliers from analysis. We categorized open-ended response data on EMAI experience and acceptability using thematic analysis.

Response rate was calculated as the number of submitted prompt response forms over the total number of prompts generated per participant.

We used log-binomial regression to estimate the association between being in the top quartile of participant response rate and participant characteristics, reporting 95% confidence intervals. Intervention and control arm participants were analyzed together. Age, the only continuous variable, was broken into three categories based on its relationship with having a response rate in the top quartile (on the log odds scale) using a LOWESS plot.

#### Response rate sensitivity analysis

Acknowledging that there were sent prompts that were not saved in the database due to data transmission errors, but were identified through the presence of submitted response form data, there may also have been sent prompts that were not saved and also did not have submitted form data. We conducted a sensitivity analysis of response rate by adding to the denominator of prompts the difference between ‘counted sent’ versus ‘expected sent’ prompts (two per day + one per week over 90 study days) per participant, assuming all of the added prompts were not responded to.

Given the focus of this analysis on implementation outcomes, the analyst was not masked to study arm allocation. Analyses were conducted using Stata 15.1 IC (StataCorp, 2018).

## Results

### Participants

**Fig 2:**
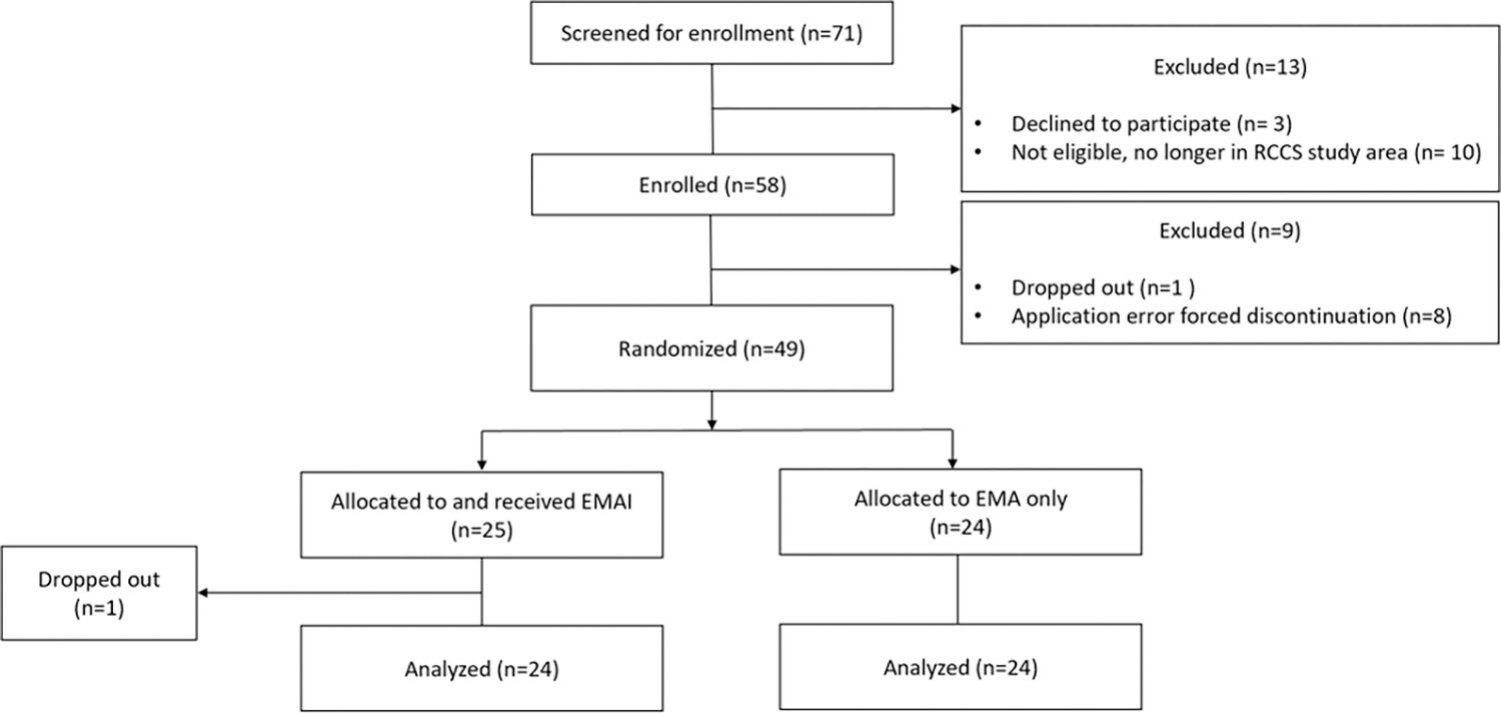
CONSORT flowchart. Figure reproduced from Beres et al, JMIR Form Res., 2021, https://doi.org/10.2196/22693.

Between 10^th^ June 2016 – 1^st^ March 2017, 71 participants were screened for enrollment, of whom 58 initially enrolled. Eight participants were excluded due to an application software error causing incorrect study arm assignments. Of the 50 fully enrolled, two dropped out at 15 days and 79-days post-enrollment, respectively. The complete analysis dataset included 48 participants, 24 per arm, a sufficient sample size with complete follow-up time to achieve pilot trial objectives (Fig 2).

Participants were 48% female with a median age of 31 years (IQR: 25–38). Everyone owned a cell phone at enrollment, with 77% reporting the ability to keep their phone charged every day, and just over half having ever used a smartphone app. At enrollment, 98% of participants reported eating fruit ≥1 day in the past month, only 6% had smoked a cigarette. (Table 1, By study arm S1 Table)

**Table 1:** Participant characteristics at enrollment.

	N	%
Total	48	100
Female	23	47.9
*Age at enrollment (years)*		
18–25	15	31.3
26–35	18	37.5
36–49	15	31.3
*Education*		
Some secondary	15	31.3
Secondary	19	39.6
Technical / Vocational	11	22.9
University	3	6.3
Own a cell phone	48	100
*Able to keep phone charged*		
Every day	37	77.1
5–6 days/week	6	12.5
3–4 days/week	5	10.4
less often	0	0
*Have cell network service in the place where you stay*		
All the time	31	64.6
More than half the time	17	35.4
Less often	0	0
Feels comfortable using a phone to send text messages	46	95.8
Knows someone who owns a smartphone	44	91.7
Ever used a smartphone app	26	54.2
**Health Behaviors, Past 30 days**	** **	** **
Smoked cigarette at least one day	3	6.3
Among smokers, days smoked at least one cigarette	min: 4, median: 20, max: 30, IQR: 4–30	
Drank alcoholic beverage at least one day	17	35.4
Among drinkers, days drank at least one alcoholic beverage	min: 1, median: 1, max: 4, IQR: 1–2	
Ate vegetables at least one day	43	89.6
Days ate at least one vegetable, among those who ate in past month	min: 1, median: 5, max: 30, IQR: 2–8	
Ate fruit at least one day	47	97.9
Days ate at least one fruit, among those who ate in past month	min: 2, median: 10, max: 30, IQR: 5–20	
Had sex with non-marital partner at least once	8	16.7
Times had sex with a non-marital or non-consensual union partner without a condom, among those engaged in behavior	min: 1, median: 2, max: 5, IQR: 1–3.5	

### Feasibility: Access to electricity and cellular reception

During the 90 days of study follow-up, 92% of participants had ≥1 day when they could not keep the phone on and charged (median: 4.5, IQR: 2–8 days). However, 90% of participants reported being able to keep the study phone on and charged for at least 85% of the study period. The majority (58%) charged their phones at home, with 19% charging at a neighbor’s home and the remainder elsewhere, such as shops or a friend’s home. One third reported there was an area where they travelled frequently that did not have cellular reception.

### Feasibility and acceptability: Response rate

#### Daily and weekly prompt response rate

Across all participants, 5,222 daily and weekly prompt response forms were submitted, 6 with missing data (0.1%). The total submitted prompt response forms, which could only be submitted in the app in response to a generated prompt, exceeded the count of sent prompts for six participants (indicating min: 1 –max: 16 known unrecorded prompts added to the ‘sent prompts’ denominator).

Per participant (N = 48), the median response rate was 66.5% (min: 30.3%, IQR: 60.0%-78.6%, max: 93.1%). The mean response rate was not a significantly different by study arm (control: 66.6%, intervention: 68.2%, p = 0.70). Across all participants, 67.4% (5222/7744) of prompts were responded to with a submitted behavioral report form.

Prior smartphone app use at baseline (aPR 3.76, 95%CI: 1.16–12.17, p = 0.03) and being in the intervention arm of the study (aPR 2.55, 95% CI: 1.01–6.44, p = 0.05) were significantly associated with being in the top response rate quartile (responding to greater than 78.6% of prompts). (Table 2)

**Table 2:** Demographic characteristics associated with being in the top response rate quartile.

	Prevalence Ratio (PR)	95% CI		p-value	Adjusted PR*	95% CI		p-value
								
**Male**	0.92	0.35	2.45	0.89	0.70	0.32	1.54	0.38
**Age group (years)**				0.38				0.18
Under 25	1.00				1.00			
25—under 35	4.00	0.56	28.73		5.96	0.99	36.02	
35+	3.20	0.41	25.00		3.78	0.56	25.63	
**Education completed**				0.18				0.94
Some secondary	1.00				1.00			
Secondary	0.79	0.19	3.37		0.83	0.29	2.35	
University or Technical / Vocational	2.14	0.66	6.97		0.89	0.30	2.63	
**Ever used a smartphone app at enrollment**	2.54	0.78	8.24	0.12	3.76	1.16	12.17	0.03
**Study arm (Intervention v. Control)**	2.00	0.69	5.76	0.20	2.55	1.01	6.44	0.05

*Sex, age, education completed, prior smartphone app use, study arm

### Sensitivity analysis: Daily and weekly prompt response rate

There was a median of 34 (IQR: 18–51) fewer counted sent prompts than expected sent prompts per participant (expected were two per day + one per week over total study days). The sensitivity analysis using expected sent prompts as the denominator showed 55.4% of prompts were responded to with a submitted prompt response form. The median per participant response rate was 58.9% (min: 22.4%, IQR: 46.9%-63.2%, max: 84.9%).

### Feasibility and acceptability: Prompt-driven behavioral self-report

#### Twice-daily prompt responses

Daily prompt-driven behavioral data were successfully collected on all topics of interest. (Table 3) During data cleaning, four quantity report outliers were excluded from analysis (<0.1%). All participants reported fruit and vegetable consumption, with only seven participants reporting cigarette smoking and 16 reporting sex with a non-marital or non-long-term partner. Condom use was reported in 38.2% of the 68 total reports of sexual encounters with non-marital or non-long-term partners (Table 3). Only one female and one person aged ≥35 years reported smoking. All other behaviors were distributed across gender and age categories (S2 Table).

**Table 3:** Behaviors reported in response to twice-daily prompts.

	Participants ever reporting behavior	Per participant reports of behavior*	Quantity per behavioral report*
Total	48	--	--
Cigarette smoking	7	1 (1–8)	2 (1–2)
		Min: 1, Max: 97	Min: 0, Max: 6
Alcohol consumption	26	3 (2–6)	Not recorded
		Min: 1, Max: 22	
Sex with a non-martial or non-long-term partner	16	3 (2–6)	n/a
		Min: 1, Max: 10	
Fruit consumption	48	72 (46–88)	1 (1–2)
		Min: 7, Max: 119	Min: 0, Max: 12
Vegetable consumption	48	47 (34–64)	1 (1–2)
		Min: 5, Max: 109	Min: 1, Max: 8

*median (IQR)

#### Weekly prompt responses

**Fig 3:**
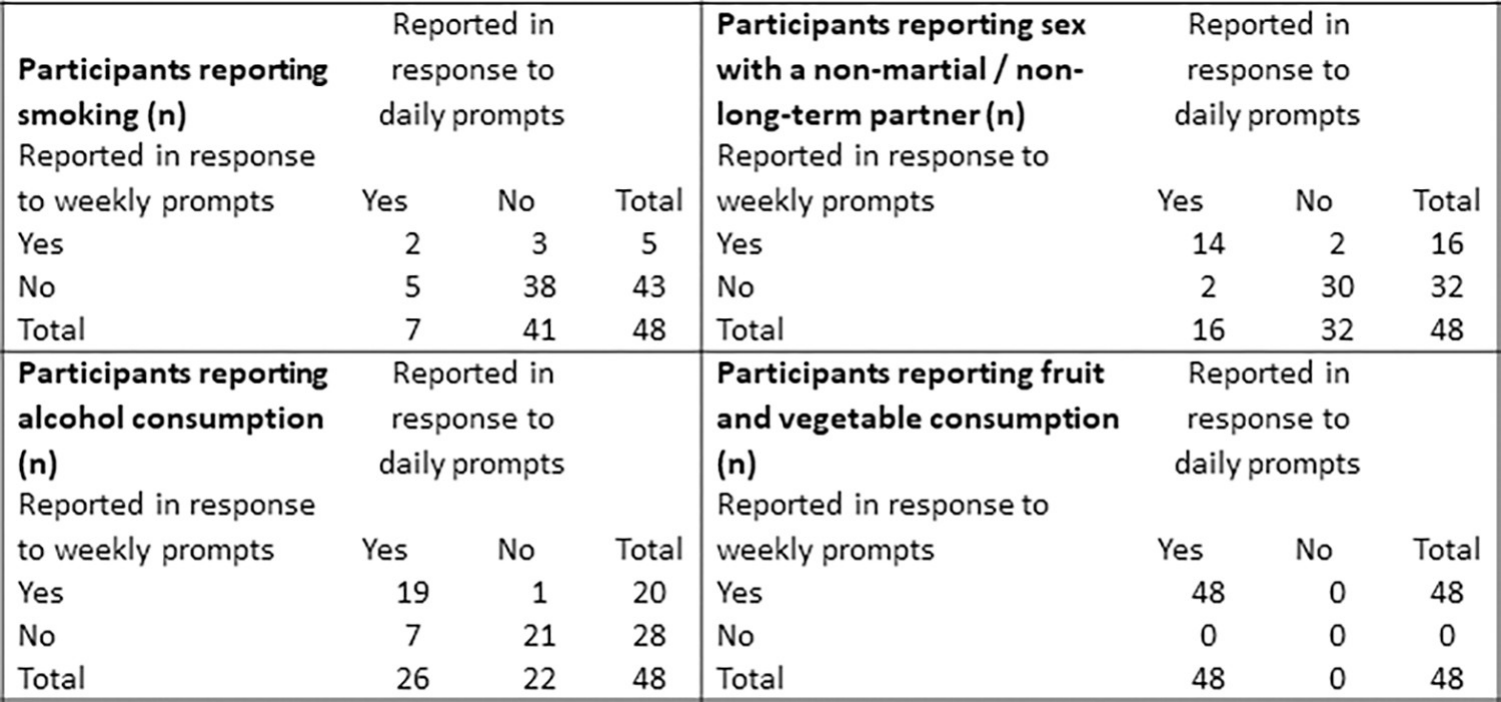
Comparison of ever reporting behavior in response to twice-daily versus weekly prompts.

Weekly prompts also successfully collected behavioral data on all topics of interest. Agreement between participants ever reporting the behaviors using daily or weekly prompts differed by question topic: percent agreement ever smoking and alcohol: 83%, ever sex with non-long-term partner: 92%; ever fruit or vegetables: 100%. (Fig 3) Frequency of behaviors was similar between reports.

#### Event-contingent participant-driven reporting

All 48 participants submitted self-initiated, event-contingent reports during the study period (submitted per participant: median: 91, IQR: 63–127). Every participant sent event contingent reports in each 30-day period of study follow-up. Of all event-contingent reports submitted, 43% were sent within the first 30 days of participant enrollment, 28% between study days 30 and 60, and 29% in the final 30 days.

**Fig 4:**
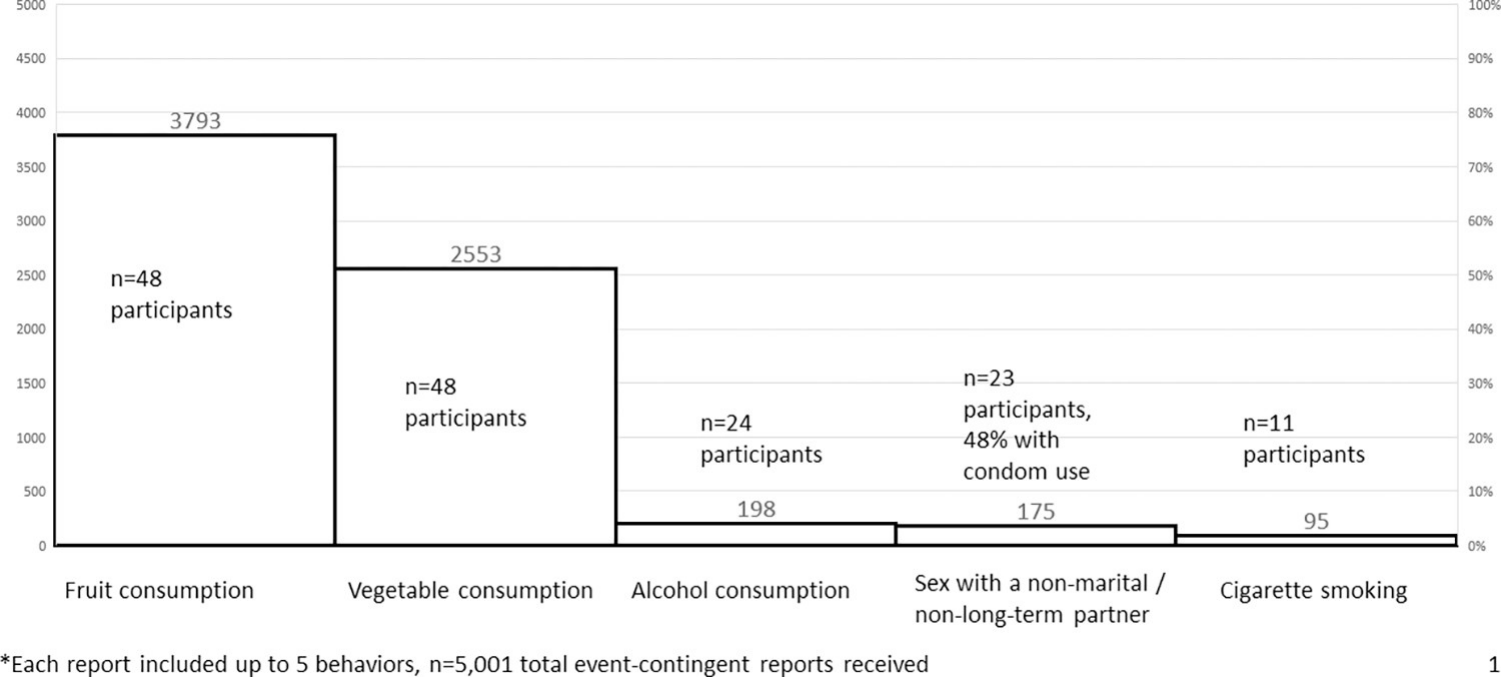
Number of event-contingent reports by behavior of interest and total participants ever reporting behavior (n)*.

Of the 5,001 event-contingent submissions received, 76% reported fruit consumption, 51% reported vegetable consumption, while ≤4% reported alcohol use, sex with a non-marital or non-long-term partner, or smoking. (Fig 4)

Approximately half, 48% (n = 23), of participants submitted at least one event contingent report of sex with a non-marital or non-long-term partner. Of the sexual encounters reported, 52% did not include condom use. Just under one quarter of participants (n = 11) reported smoking, half (n = 24) reported alcohol consumption, and all (n = 48) reported fruit and vegetable consumption. Average number of reported cigarettes smoked in the hour prior to submission was 1.5 (min: 0, max: 6). Average consumption of vegetables and fruit reported was 1.5 (min: 0, max: 11) and 1.4 (min: 1, max: 14), respectively. During data cleaning, nine quantity report outliers were excluded from event contingent reporting data (<0.2%).

### Feasibility: Geospatial data collection

The study collected a median of 2,509 momentary geospatial coordinate reports per participant (min: 68, max: 7,538 IQR:1,339–3,420). Across all participants, geospatial coordinate reports were collected on an average of 71 of the 90 study days. All participants had at least one gap of >24 hours between coordinate collection, with 22/48 experiencing a gap of a week or greater (max: 32 days).

### Feasibility: Reported phone and app use

**Fig 5:**
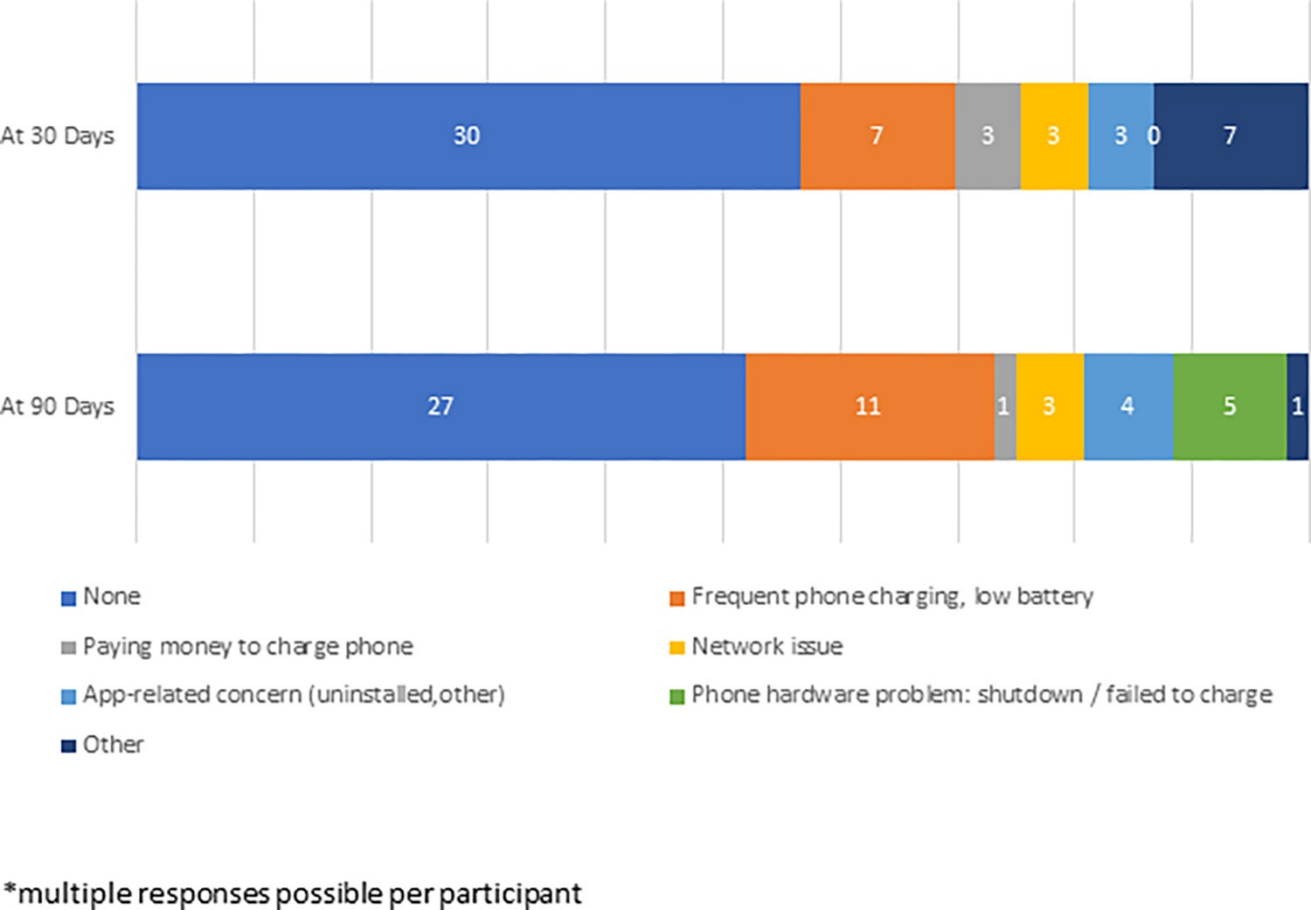
Participant-reported phone problems*.

At study end, 56% of participants reported no problems with their smartphones. Approximately one-quarter reported challenges related to the need for frequent charging, with 10% reporting a phone hardware failure (shutting down or failing to charge). (Fig 5) No study phones were broken nor lost. Individual participants discussed app use challenges (e.g. selecting a response in error, difficulty initial remembering their security pin code).

### Acceptability: Perceptions of EMAI participation

When asked study aspects they disliked, 63% of all 48 participants who completed exit questionnaires at day 90 reported no dislikes. Approximately one quarter reported not liking questions about smoking, with most indicating it was because they did not smoke and/or did not like smoking. Others raised negative perceptions of alcohol use and sexual intercourse questions. Several participants reported disliking an aspect of the app set-up, including receiving alerts at night and length of time taken to open the app.

In response to what they liked, one-third of participants mentioned sending event-contingent reports. Nearly one-fifth commented positively on receiving daily prompts. Each question topic was mentioned with positive regard by several to half of participants. Many participants mentioned they valued receiving health messages. All participants reported they would sign up to receive reminders on their personal phone after the study ended.

## Discussion

Study data demonstrate that mobile EMA data collection and real-time, responsive health messaging are feasible and acceptable in Rakai, Uganda. Our study showed high participant retention among a demographically varied participant group, as well as successful collection of both prompt and participant-driven behavioral data throughout the study period. Intervention arm allocation was significantly associated with the top response rate quartile (responding to > 78% of prompts), indicating that behaviorally-responsive messaging was desirable. Smartphone app use prior to study start was also associated with response rate, suggesting that EMAI use in the general population may increase as smartphone use becomes more common. Collected behavioral data are broadly consistent with regional estimates [18, 19] and baseline reports, although risk behaviors are more prevalent in EMA data as may be expected of event-based collection [1], demonstrating plausibility of reported data. Most participants reported positive experiences of study participation, including message sending and the question content, consistent with studies in high-income settings across a broad range of participant populations and content areas [20–24]. Since our study period, the Ugandan GSMA connectivity index increased from 34 in 2016 to 40 in 2019, suggesting broader access within the general population and an environment increasingly well-suited to mobile data collection [25].

While half of participants had a response rate of sixty percent or greater, efforts may be needed to improve prompt-driven EMA response. Our study used training, participant follow-up and financial incentives to support data submission, but saw wide variation in response rate. Our findings are consistent with other EMA research, demonstrating response rates ranging from 31–90%, inclusive of studies incentivizing prompt-based responses [14, 26–28]. While limited by the small pilot sample size, our data suggest that greater familiarity with smartphone applications at enrollment may be associated with EMA response success. This may recommend additional training for smartphone naïve participants and indicate further mobile EMAI potential as personal smartphone use expands. To improve response rates, extant literature supports tailored design of questions, prompt timing, and response mechanisms for a study population, highlighting the importance of pilot testing [14, 28, 29]. Participants generally understood questions well, submitting <0.2% implausibly large values or values of ‘0’ for quantity of a reported behavior.

Data management is of critical importance for EMAI studies. Analysis of behavioral report forms demonstrated that not all prompts were recorded in the study database, likely due to challenges with data transmission. Comparing data stored on the phone itself to the study database periodically throughout data collection, or combining prompt and response data processing tasks may improve data completeness in future studies.

Despite the likely influence of social desirability bias on risk behavior reports, approximately half of study participants reported behaviors including smoking, alcohol consumption and sex with a non-long-term partner without being prompted, demonstrating the feasibility of using event-contingent EMAI for data collection and intervention on potentially sensitive topics. This is consistent with our evidence-driven hypothesis that participant-led, remote data collection should minimize under-reporting across question topics [7, 9, 23, 24] and the theoretical grounding [1] of EMA as a tool for behavioral data collection. However, future research comparing EMAI with in-person interview responses would further inform EMAI application. Negative reactions to specific question topics elicited through open-ended participant, however, suggest that interventional studies may need to target topics based on behavioral prevalence or other participant characteristics.

All participants in our study had data collection gaps of at least a day with 46% experiencing a gap of a week of more. While these gaps were not explicitly explained, device power-related obstacles were the most commonly reported study challenges. Future EMAI studies in similar settings using mobile technology should consider supporting battery-charging and extended power supplies to maximize feasibility. Studies should anticipate that, while overall device access can remain high, many participants will likely experience episodic access barriers due to lack of battery, network availability, or other reasons. Study power calculations will need to plan for this likely episodic non-response or non-availability of geospatial coordinate collection. Consistent with other studies using mobile technology in low-resource settings [30], while creating challenges for less than 10% of the EMAI participants, studies need infrastructure for participant training and to troubleshoot app and hardware concerns.

### Limitations

As a pilot, the findings are not clearly generalizable beyond our study setting. However, they may apply to transferable settings and offer proof of concept suggesting the appropriateness of future research. The study was conducted among an established cohort. Their successful participation in other survey research may influence their ability to participate in our trial. Our study follow-up period was limited to 90-days and up to 10 simultaneous participants. While study procedures and participation were consistent over this period, EMAI work of extended duration and participant sample size may require additional measures and revised procedures to support valid and reliable implementation [14].

## Conclusions

EMAI using mobile technology is a feasible and acceptable technique that may support improved behavioral data collection and intervention approaches. This novel approach should be tested in larger studies. Future studies should also compare EMA data with other collection methods in LMIC populations and trial the effect of EMAI on behavior change.

## Supporting information

S1 Checklist Inclusivity in global research checklist. (DOCX)

S2 Checklist Study CONSORT checklist. (DOC)

S1 Table Participant characteristics by study arm. (DOCX)

S2 Table Sex and age distribution of risk behaviors reported in. (DOCX)

S1 Appendix Ecological momentary assessment questionnaires. (DOCX)

S1 Protocol Study protocol: Mobile behavioral ecological momentary assessment and intervention in Rakai, Uganda. (PDF)
