# Supplementary material for: Acceptability and feasibility of mobile phone-based ecological momentary assessment and intervention in Uganda: A pilot randomized controlled trial
Source: PLoS One. 2022 Aug 26;17(8):e0273228. doi: 10.1371/journal.pone.0273228 (PMC9416993; doi:10.1371/journal.pone.0273228)
Supplement: S1 Table — (DOCX) [file pone.0273228.s003.docx]

**Supporting Information 2 Table. Participant characteristics by study arm**

| **Participant characteristics** | | **Control (n=24)** | **Intervention (n=24)** | **Total (N=48)** | ***P* value^a^** |
| --- | --- | --- | --- | --- | --- |
| Female, n (%) | | 12 (50) | 11 (46) | 23 (48) | .77 |
| Age at enrollment (years), mean (SD) | | 32.7 (7.1) | 30.1 (6.7) | 31.4 (7.0) | .10 |
| **Education completed, n (%)** | | | | | .94 |
|  | Some secondary | 7 (29) | 8 (33) | 15 (31) |  |
|  | Secondary | 10 (42) | 9 (38) | 19 (40) |  |
|  | University, technical or vocational | 7 (29) | 7 (29) | 14 (29) |  |
| **Yes, owns a cell phone, n (%)** | | **24 (100)** | **24 (100)** | **48 (100)** | **—** |
| **Able to keep phone charged, n (%)** | |  |  | 48 (100) | 0.21 |
|  | Every day | 16 (67) | 21 (88) | 37 (77) |  |
|  | 5-6 days/week | 4 (17) | 2 (8) | 6 (13) |  |
|  | 3-4 days/week | 4 (17) | 1 (4) | 5 (10) |  |
|  | Less often | 0 (0) | 0 (0) | 0 (0) |  |
| **Have cell network service in the place where you stay, n (%)** | |  |  | 48 (100) | 0.76 |
|  | All the time | 15 (63) | 16 (67) | 31 (65) |  |
|  | More than half the time | 9 (38) | 8 (33) | 17 (35) |  |
|  | Less often | 0 (0) | 0 (0) | 0 (0) |  |
| Yes, feels comfortable using a phone to send text messages, n (%) | | 24 (100) | 22 (92) | 46 (96) | .15 |
| Yes, knows someone who owns a smartphone | | 23 (96) | 21 (88) | 44 (92) | .30 |
| Yes, ever used a smartphone app, n (%) | | 14 (58) | 12 (50) | 26 (54) | .56 |
| **Occupation, n (%)** | | | | | .53 |
|  | Agrarian | 6 (25) | 8 (33) | 14 (29) |  |
|  | Trader | 5 (20) | 3 (12) | 8 (16) |  |
|  | Teacher | 4 (17) | 7 (29) | 11 (23) |  |
|  | Other | 9 (38) | 6 (25) | 15 (31) |  |
| **Health behaviors, past 30 days** | | | | | |
|  | Smoked cigarette at least one day, n (%) | 3 (13) | 0 (0) | 3 (13) | .07 |
|  | Among smokers, days smoked at least one cigarette, mean (SD) | 20 (13.1) | — | — | — |
|  | Drank alcoholic beverage at least one day, n (%) | 8 (33) | 9 (38) | 17 (35) | .76 |
|  | Among drinkers, days drank at least one alcoholic beverage, mean (SD) | 2.1 (1.3) | 1.6 (0.7) | — | .29 |
|  | Ate vegetables at least one day, n (%) | 22 (92) | 21 (88) | 43 (90) | .64 |
|  | Among those who ate vegetables, days ate at least one vegetable, mean (SD) | 7.2 (5.4) | 6.7 (7.9) | — | .80 |
|  | Ate fruit at least one day, n (%) | 23 (96) | 24 (100) | 47 (98) | .31 |
|  | Among those who ate fruit, days ate at least one fruit, mean (SD) | 13.6 (9.0) | 12.5 (8.2) | — | .67 |
|  | Had sex with nonmarital or nonlong-term partner without using a condom at least once, n (%) | 5 (21) | 3 (13) | 8 (17) | .44 |
|  | Times had sex with a nonmarital or nonlong-term partner without a condom, among those reporting sex, mean (SD) | 2.4 (1.9) | 2.3 (0.6) | — | .96 |

a Two-sided P value calculated using chi-square tests for categorical variables and Student t test for continuous variables.
