# Supplementary material for: Acceptability and feasibility of mobile phone-based ecological momentary assessment and intervention in Uganda: A pilot randomized controlled trial
Source: PLoS One. 2022 Aug 26;17(8):e0273228. doi: 10.1371/journal.pone.0273228 (PMC9416993; doi:10.1371/journal.pone.0273228)
Supplement: S2 Table — (DOCX) [file pone.0273228.s004.docx]

**Supporting Information 3 Table. Sex and age distribution of risk behaviors reported in response to twice-daily prompts.**

|  | **Participants ever reporting behavior** | **Per participant reports of behavior*** |
| --- | --- | --- |
| **Total** | **48** | **--** |
| **Cigarette smoking, total** | 7 | 1 (1-8), Min: 1, Max: 97 |
| Male | 6 | 1.5 (1-8), Min: 1, Max: 97 |
| Female | 1 | 1 |
| Under 25 years | 3 | 1 (1-97), Min: 1, Max: 97 |
| 25 – under 35 years | 3 | 2 (1-8), Min: 1, Max: 8 |
| 35 years+ | 1 | 1 |
| **Alcohol consumption, total** | 26 | 3 (2-6), Min: 1, Max: 22 |
| Male | 13 | 3 (2-7), Min: 1, Max: 20 |
| Female | 13 | 3 (2-4), Min: 1, Max: 22 |
| Under 25 years | 4 | 4 (3-5.5), Min: 3, Max: 6 |
| 25 – under 35 years | 14 | 2 (1-8), Min: 1, Max: 22 |
| 35 years+ | 8 | 3 (1.5-5.5), Min: 1, Max: 20 |
| **Sex with a non-martial or non-long-term partner** | 16 | 3 (2-6), Min: 1, Max: 10 |
| Male | 10 | 3.5 (2-6), Min: 2, Max: 10 |
| Female | 6 | 2 (1-10), Min: 1, Max: 10 |
| Under 25 years | 5 | 2 (2-3), Min: 2, Max: 4 |
| 25 – under 35 years | 6 | 5 (2-10), Min: 1, Max: 10 |
| 35 years+ | 5 | 2 (2-6), Min: 1, Max: 10 |

*median (IQR)
