# Supplementary material for: Acceptability and feasibility of mobile phone-based ecological momentary assessment and intervention in Uganda: A pilot randomized controlled trial
Source: PLoS One. 2022 Aug 26;17(8):e0273228. doi: 10.1371/journal.pone.0273228 (PMC9416993; doi:10.1371/journal.pone.0273228)
Supplement: S1 Appendix — (DOCX) [file pone.0273228.s005.docx]

**Supporting Information 4. Ecological Momentary Assessment Questionnaires**

| **Daily Prompt-Driven Response Questionnaire** |
| --- |
| Did you smoke today? |
| How many cigarettes did you smoke today? |
| Did you eat any fruits today? |
| How many fruits did you eat today? |
| Did you eat any vegetables today? |
| How many vegetables did you eat today? |
| Did you drink alcohol today? |
| Did you have sex with someone that is not a long-term/marital partner? |
| Did you use a condom each time you had sex with someone other than your long-term/marital partner today? |
| Thank you for your time! |
|  |
| **Weekly Prompt-Driven Response Questionnaire** |
| Did you smoke in the past week? |
| How many days last week did you smoke? |
| Approximatly how many cigarettes did you smoke each day last week that you smoked? |
| How many days last week did you eat fruits? |
| Approximately how many fruits did you eat each day that you ate fruit? |
| How many days last week did you eat vegetables? |
| Approximately how many vegetables did you eat each day that you ate vegetables? |
| Did you drink alcohol last week? |
| How many days last week did you drink alcohol? |
| Did you have sex with someone that is not a long-term/marital partner last week? |
| Did you use a condom each time you had sex with someone other than your long-term/marital partner last week? |
| Thank you for your time! |
|  |
| **Event Contingent Response Questionnaire** |
| In the past hour, which of the following did you do? Check all that apply. |
| Smoked |
| Drank alcohol |
| Had sex without a condom with someone who is not a long-term/marital partner |
| Ate fruits |
| Ate vegetables |
| How many cigarettes did you smoke in the past hour? |
| Did you use a condom? |
| How many fruits did you eat? |
| How many vegetables did you eat? |
| Thank you for your time! |
