# Supplementary material for: Acceptability and feasibility of mobile phone-based ecological momentary assessment and intervention in Uganda: A pilot randomized controlled trial
Source: PLoS One. 2022 Aug 26;17(8):e0273228. doi: 10.1371/journal.pone.0273228 (PMC9416993; doi:10.1371/journal.pone.0273228)
Supplement: S1 Protocol — (PDF) [file pone.0273228.s006.pdf]

Study Protocol:
Mobile Behavioral Ecological Momentary Assessment and Intervention in Rakai, Uganda

Protocol Date: 30th March 2015

ClinicalTrials.gov Identifier: NCT04375423

JHM IRB - eForm A – Protocol

****

Mobile Behavioral Ecological Momentary Assessment and Intervention in Rakai, Uganda: A Pilot Study

1. Abstract

Ecological Momentary Assessment and Intervention (EMAI) is an emerging technique for gathering richer and more relevant data through repeated, longitudinal sampling of participants in their natural setting in order to deliver real-time interventions. Challenges with instilling healthy behaviors are well documented. These challenges are magnified in low and middle income countries (LMICs) where significant social and structural barriers to care often exist. The rapid emergence of mHealth (mobile technologies for health), including growing access to low-cost smartphones, now allows for unprecedented advancements in and novel research on mHealth applications in LMICs. We therefore propose a pilot EMAI study of health behavior in Rakai, Uganda.

This study aims to demonstrate that it is feasible and acceptable to use smartphones in a LMIC to collect near real-time, geolocated behavioral information and to send tailored health messages based upon this data. This proposal will provide unique insights into the geospatial/temporal epidemiology of health behaviors, generate preliminary estimates of intervention impact, and establish an evidence-base for future large-scale, randomized EMAI mHealth trials. This study will be nested within the Rakai Health Sciences Program's (RHSP) ongoing Rakai Community Cohort Study (RCCS).

Our main study objective is to conduct a pilot EMAI study among current RCCS study participants. Participants will be given a smartphone with an application that will collect GPS-based geolocations and ask assessment questions (e.g. diet/alcohol, smoking, and sexual behaviors). Using participants' responses, the application can deliver tailored intervention health messaging to participants using pre-programmed algorithms. During Days 1-30 of follow-up, all participants will receive assessment questions only to establish baseline behaviors. During Days 31-90, participants will be randomized to either continuing with assessment questions only (control arm) or to also begin receiving intervention messaging (intervention arm). The randomized design will allow for calculation of an estimate of EMAI effect on diet/alcohol, smoking, and sexual behaviors which will be used to design future, large-scale randomized controlled trials of EMAI.

Secondary objectives of this study proposal are to assess processes, facilitators, and barriers to EMAI using a mixed methods (quantitative and qualitative) approach. Also, to assess EMAI validity and relevance, we will describe and compare EMAI-collected behavioral data with traditional questionnaire-collected data.

Study hypotheses are as follows: **EMAI can be successfully implemented in Uganda, and participants receiving intervention messaging will have improved self-reported health behaviors compared to controls; EMAI will be feasible and acceptable by this population; and, EMAI-collected data will correlate with traditional questionnaire-collected data.**

This is the first study to our knowledge of EMAI in sub-Saharan Africa. If this novel data collection and intervention method is proven to be robust, feasible and acceptable, it could serve as an innovative platform for the future delivery of real-time, tailored, geolocated mHealth interventions, a potentially profound method for improving health.

2. Objectives

Primary Objective: To conduct a pilot EMAI study in Rakai, Uganda.

Secondary Objective: To assess processes, facilitators, and barriers to EMAI.

Secondary Objective: To compare EMAI-collected with traditional questionnaire-collected data.

3. Background

Smartphone-based mHealth is a potentially transformative technology in low and middle-income countries (LMIC). The growth of mobile phones in LMIC has been remarkable.[1] Initial mobile growth primarily began with "dumb" or feature phones, i.e. mobile devices with limited features and internet connectivity. These devices were rapidly leveraged for health care purposes as part of a "first wave" of applications based primarily on SMS (short message service) technology.[2,3] Mobile infrastructure has progressively expanded in LMICs and more recently, smartphones have been steadily increasing in number with an estimated 19.8% penetration in 2013, up from 1.6% in 2008.[4] With the increasing adoption of smartphones in LMICs, a "second wave" of mHealth innovation based upon networked, data-driven applications is possible.

EMAI may outperform traditional methods of data collection and behavioral intervention. Ecological Momentary Assessment and Intervention (EMAI) is an emerging technique for gathering richer and more relevant data through repeated, longitudinal sampling of participants and intervening in real-time in their *natural setting*.[5] Compared to traditional assessment of human behavior, e.g. questionnaires and interviews, Ecological Momentary Assessment (EMA) appears to reduce recall bias, improve validity by collecting data in the real world, and enable capture of dynamic processes over time,[5] which has also been supported by our own studies which have collected reliable data on geo-located, sensitive behaviors such as drug use, mood, alcohol intake, and sexual behaviors.[6-8] Ecological Momentary Intervention (EMI) has benefits beyond traditional interventions by allowing for more temporally and spatially appropriate interventions, extending care beyond traditional health facilities and into the individual's natural setting. Smartphones have revolutionized access to EMAI and provide a growing platform for health-related assessment and intervention in LMICs.

A disease and prevention/treatment agnostic EMAI platform could be highly scalable. This study proposes to test a disease and prevention/treatment agnostic EMAI platform. We will test this platform by targeting three behavioral areas of high public health significance, i.e. smoking, diet/alcohol, and sexual activity, which contribute to smoking-related cancers, obesity, and HIV transmission. This agnostic platform could be adapted for most health care conditions, making it highly scalable and increasing potential impact.

Preliminary studies. Our group has done extensive and pioneering work in mHealth (including EMAI), mixed methods research, and longitudinal surveillance of health behaviors. *emocha*-We have experience in the design, development, implementation, and evaluation of mHealth as founders of *emocha*,[10-12] an mHealth software platform. We have conducted a number of EMAI studies domestically, including The EXposure Assessment in Current Time (EXACT) study (5U01DA023832; PI: Gregory Kirk) assessed drug use and psychosocial stress in injection drug users (IDUs) in urban Baltimore. We developed a EMA application for this study which was successfully implemented with excellent participant response and demonstrated validity.[6,7] Another EMAI application developed by

our group to assess HIV care engagement (clinic attendance and adherence), drug cravings, and psychosocial stress to improve retention in care and adherence to medications for out-of-care, HIV-infected IDUs has also been successfully implemented (R34DA033181; PIs: Kirk, Westergaard). Finally, we have developed an EMA application to assess alcohol use and sexual behaviors in men who have sex with men in Baltimore which successfully recruited participants (P30AI094189; PI: Cui Yang).

4. Study Procedures

<u>Primary Objective: To conduct a pilot EMAI study with smartphones in Rakai, Uganda.</u>

Study design. This pilot study will be nested within the Rakai Community Cohort Study (RCCS, WIRB #20031318), an ongoing, population-based, prospective, observational study which surveys Rakai District communities in Uganda every ~12-18 months.

Study setting and population. Rakai District (~2200 km^2) is a mostly rural district (population~400,000) in Uganda bordered by Tanzania and Lake Victoria. RHSP has been conducting community-based research in Rakai since 1988 including large, prospective health behavior studies.[26] RHSP survey results of demographic and behavioral characteristics are similar to those reported for southwestern Uganda in Demographic and Health Surveys,[27] suggesting that despite long-term surveillance and health education, the population is typical of rural/semi-rural Uganda.[28]

<u>Rakai Community Cohort Study (RCCS):</u> RHSP's ongoing 54 community (n~20,000) longitudinal HIV surveillance study provides >20 years of data on HIV prevalence/incidence, health behaviors, sexual networks, and mobility/migration in Rakai District, Uganda.

Eligibility. We will aim to recruit 120 total adults (age >=18 years) from current, literate RCCS study participants.[29] We will purposely recruit a sample that has a broad range of participant characteristics in our study to optimize diversity of responses and increase study generalizability. Specific variables for which we will target sampling include: gender (~50% female), age (at least 20% among age groups 18-25, 26-35, >50), and occupation (at least 20% traders and farmers).

Recruitment. As part of a detailed written informed procedure, RCCS participants have consented to be contacted for future studies. We will enumerate a list of current RCCS participants with key demographics, and, as described above, we will purposively select participants meeting our study population composition goals. We will begin with recruitment via RCCS in locations where it is actively conducting its continual surveys of the Rakai community, asking participants as they undergo the RCCS survey if they would also like to participate in this nested study. If we are unable to conveniently obtain our desired sample via ongoing survey activities of RCCS, we will send trained staff to recruit RCCS participants from communities in which RCCS has recently conducted surveys. Eligible participants will be asked to complete a written informed consent procedure (see Section 8 below) in order to enter this specific study.

Participant-related Procedures. *Participant training.* After recruitment, participants will undergo a ~30-60 minute training session. A brief training curriculum will be used consisting of basic phone use,

contacting the study team, and detailed instruction on EMAI application use. Participants will be required to demonstrate proficiency with the EMAI interface prior to receiving a study smartphone. The first 48 hours after the participant has the phone will be a "run-in" period to allow for troubleshooting of device problems and to allow participants to become familiar with the EMAI application procedures.

Follow-up (Days 1-30, Baseline Behaviors). After the run-in period, all participants will receive assessment questions only for a 30 day period in order to establish baseline behaviors. After completing this initial 30 day period, participants will be required to return to the study office to complete a short questionnaire (see Aim 3) and to be randomized to the second phase of follow-up.

Follow-up (Days 31-90, Randomized Evaluation). After completing the Day 30 questionnaire, participants will be randomized using a computerized random number generator, in a 1:1 ratio, to either continue receiving assessment questions only (control arm) or to also begin receiving intervention messages (intervention arm). After reaching 60 and 90 days of follow-up, participants will return to the study office to complete a brief questionnaire on behaviors and experiences with the phone. After 90 days of follow-up participants will also return their phones to study staff.

Logistics. Phones will be preloaded with airtime. If additional airtime is needed, participants can "beep" (call and hang up, no cost to caller) study staff to transfer airtime to phones using locally available services.

Smartphone loss/theft. Installed software will allow for GPS-locating of lost/stolen phones and can remotely wipe data on phones as needed. Participants will be notified that they will not be eligible for their incentives if they lose or have their phones stolen. These participants will remain in all study analyses (intention-to-treat) but will not receive a second phone.

Smartphone malfunction. Participants will be asked to communicate with study staff either in-person or remotely should their smartphone malfunction. Malfunctioning phones will be returned to study staff if necessary. IT support will be available to troubleshoot phones during regular business hours throughout the study. Study staff will record all instances of malfunctioning phones as part of process evaluations.

EMAI Application. The EMAI application for this study will be programmed following user centered design principles on the *emocha* platform.[30]

Prompts. The EMAI application will send prompts (audible alert and text message asking them to complete a specific form, no specific health information revealed in prompts) to participants at 3 times: daily, random, weekly. Daily prompts will occur once a day at a scheduled time, a random prompt occurs once a day at an unscheduled time, and a weekly prompt occurs once a week at a scheduled time. The diversity of prompts allows for a range of response patterns.[5] Participants will be allowed to respond to read form and respond to prompts only after entering a password.

Event-contingent reporting. In addition to prompts, participants will also be able to report behaviors on an event-contingent basis, i.e. within an hour after they engage in a specific behavior. A password will again be required to input data.

Data collection forms. The EMAI application will automatically launch the appropriate password-protected forms with each prompt, or they can be launched by the participant for event-contingent reporting. Forms will be completed using touchscreen input. Simple yes/no and multiple-choice check list questions will be used. Forms are designed to be typically completed in <1 minute. Specific form content is shown in Table 1.

Geolocation. The EMAI application will automatically record participant geolocation via GPS approximately hourly and each time a form is completed.

Data transfer. All participant-entered and geolocated data will be initially stored in encrypted format on the phones. When cellular network or WiFi coverage is detected by the application, the data will be synced to a server for study staff to monitor responses. Updates to forms and algorithms will be sent from the server to phones as needed.

Table 1: EMA application sample questions.

<table>
<tr><th>Forms</th><th>Behavior Domains</th><th>Questions</th></tr>
<tr><td>Daily</td><td rowspan="3">Smoking, Diet/Alcohol Use, Sexual Activity</td><td rowspan="3">Did you smoke today/this week? (yes/no) If so, how many items did you smoke today? (#); Did you eat any vegetables today/this week? (yes/no); Did you use any alcohol today/this week? (yes/no); Did you have any sex with anyone who was not your marital or long-term partner today/this week? (yes/no) If so, did you use condoms? (yes/no)</td></tr>
<tr><td>Random</td></tr>
<tr><td>Weekly</td></tr>
<tr><td>Event-contingent</td><td>Smoking, Diet/Alcohol Use, Sexual Activity</td><td>Checklist Format: Within the past hour, I smoked, ate vegetables, drank alcohol and/or had sex with someone who was not my marital or long-term partner.</td></tr>
</table>

In response to data entered by participants, the EMAI application will be programmed with algorithms to provide real-time feedback, i.e. EMI, through tailored health text messages. Only patients randomized to the EMI arm will receive these messages. We will use general health as well as behavior-specific messaging. Examples are shown in Table 2.

Table 2: Examples of EMI responses.

EMA Data Collected	EMI Response
Any "negative" health behavior entered.	*"Today is a great day to start being healthy." "You can make a difference in your health." "You have people around you who care about you."*
Did you eat any vegetables? **NO**	*"Eating a mixed diet (meats and vegetables) is a healthy way to go."*
Participant enters a geolocation previously associated with smoking.	*"There are better things to spend money on than cigarettes." "Smoking causes lung cancer."*

Cultural Adaption, Language, and Literacy Formative Work. Prior to beginning formal recruitment, we will test the appropriateness of our questions and messaging in terms of cultural adaptation, clarity, and meeting literacy requirements. Specifically, we will use our initial list of proposed questions and messages and then ask potential participants (~10) in the study whether the questions and messaging are clear, acceptable, and correctly understood by them. We will then refine questions and messaging as needed.

Secondary Objective: To assess processes, facilitators, and barriers to EMAI-These results will inform future EMAI implementation, scale-up, and sustainability questions. We will utilize a mixed methods (quantitative and qualitative) approach involving process indicator analyses, in-depth interviews (IDI), and focus group discussions (FGD).[33]

Quantitative Methods and Analytic Plan for Process Measures. We will gather process data during EMAI implementation to include: *EMA prompt response rate, form completion rates, out-of-network time, malfunctions, down time,* and *device retention*. Process measures analyses will be exploratory in nature. We will use descriptive statistics, as well as explore associations with moderating/mediating

factors such as gender and age using Chi-square, Fisher's exact test, and multivariate regression methods as appropriate.

Qualitative Methods. *Study Population.* All participants will take part in IDIs (n=120) which will explore individual experiences with the EMAI application. IDIs will occur immediately after completion of the 90 day follow-up period. Smaller groups (6-8 each) will participate in 2 FGDs after Day 30 and Day 90 of the study (4 FGDs total, ~24-32 participants total) to create a more dynamic and interactive forum for additional insights. *Data Collection.* RHSP has an experienced Qualitative Research Department to help ensure high quality data collection.[34-36] Semi-structured interview guides will be used for qualitative data collection with open-ended questions on EMAI processes, barriers, facilitators, confidentiality, satisfaction, challenges, suggestions for improvement, and perceived effect of EMI on behaviors. We will also explore issues regarding long-term adoption of this type of technology, gender-specific considerations, and how EMAI is or is not integrated in relation to current everyday use of mobile phones in this setting. All IDIs and FGDs will be conducted in the participants' preferred language and digitally recorded. Translation and transcription will be done in a single step by trained staff. *Analytic Plan.* Data will be entered into ATLAS.ti software (GmbH, Germany) to facilitate coding and analysis. Following an iterative approach, transcript review will begin while IDIs and FGDs are still underway and participant responses will be used to shape questions for future interviews. Initial line-by-line coding will be completed on a sample of transcripts to develop a codebook, which will then be used to categorize responses into thematic categories. Quotes from coded transcripts will be triangulated with the original transcripts to ensure appropriate contextualization. The study team will meet and discuss identified themes and come to consensus on what modifications, if any, need to be incorporated into the EMAI application in future iterations. These modifications will be carefully documented.

Secondary Objective: To compare EMAI-collected with traditional questionnaire-collected data- We will describe and compare EMAI versus traditional questionnaire-collected data.

Methods. All study participants will be administered a traditional paper-based questionnaire at the completion of the 30 day follow-up period and a similar questionnaire after the 60 and 90 day follow-up periods. This questionnaire will assess satisfaction and acceptability of the participant's experience using EMAI. Additionally, the questionnaire will ask the same behavioral questions as the EMAI application, but ask participants to recall these behaviors over the past 30 days, e.g. over the past 30 days, how many days did you drink alcohol?

Analytic Plan. Responses to traditional questionnaire data will be compared with EMAI collected data using descriptive statistics such as percent agreement, Kappa statistic, scatter plot correlation graphs, mean within-pair differences, coefficient of variation, and Bland-Altman plots.[37] Successful completion of this aim will generate important preliminary evidence on EMAI acceptability and reliability to support future NIH studies.

Timeline for proposed study.

**Activity** **Study Month→**	1-2	3-4	5-6	6-7	7-8	9-10	11-12	13-14	15-16	17-18	19-20	21-22	23-24
Formative work on messaging	■												
mHealth EMAI Software Programming and Testing	■	■											
EMAI Recruitment and Implementation (90 days total per participant, follow-up periods will be asynchronous)			■	■	■	■	■	■	■	■			
Mixed Methods Evaluations			■	■	■	■	■	■	■	■	■		
Final Analyses and Dissemination											■	■	■

5. **Inclusion/Exclusion Criteria-**See above.

6. **Drugs/ Substances/ Devices**-Not applicable.

7. **Study Statistics (for Primary Objective)**

Sample Size/Power Calculations. This is an exploratory study powered not to assess intervention efficacy but to provide preliminary estimates of effect. As an example, Table 3 shows power calculations for alcohol use based on different estimates of intervention impact. Prior data indicate that approximately 50% of the Rakai population uses alcohol on a weekly basis. If there is a 50% reduction in alcohol use following EMAI, the study will have 81.5% percent power to detect this difference.

Table 3: Power calculations.

% Reduction In Behavior	Power
20	0.195
30	0.381
40	0.611
50	0.815

Change in health behaviors following EMAI. We hypothesize that EMAI results in improved health behaviors. We will use data on health behaviors obtained prior to the start of the randomized phase, Days 1-30, to characterize baseline behaviors for each participant. Changes in baseline behaviors will be assessed from 30-90 days relative to baseline in intervention and control arms. The relative change from baseline to follow-up (e.g. change in proportion of yes responses to daily alcohol use over Days 1-30 compared to Days 31-90), will be compared between intervention and controls arms using mixed effects models. Temporal trends in behaviors in each arm over the entire 90 day period also will be identified and characterized.

Mobility patterns and health outcomes. The extent to which individuals travel local and long distances is likely a significant predictor of health outcomes We will use EMAI data to characterize participants' mobility patterns and the associations between these patterns and health behaviors in intervention and control arms.[31] All GPS data will be cleaned using standardized methods,[32] with emphasis on identification in coordinate outliers and missing coordinate data. Individuals in each arm will be classified into 3 groups based on their mobility data: low mobility (limited local movement), high local mobility (frequent local movement only), and high long distance mobility (frequent local and longer distance travel or long distance travel only). Spatial travel kernels at daily, weekly, and monthly intervals will be estimated using maximum likelihood methods and GPS data fit to various statistical distributions. The best fit spatial kernels will be used to determine the extent of local and longer distance movement and to establish cutoffs for the appropriate categorization of mobility patterns, where individuals' primary residential location will serve as the anchor location from which distance of movements are measured. Mobility patterns will be correlated with participant demographics and self-report of behaviors obtained via the EMAI using random effects regression models for binary and ordinal outcomes in intervention and control arms. Data on mobility patterns and their association with health outcomes will be informative to health behavior programs and for a wide variety of modelling studies on infectious disease transmission, including HIV.

8. **Risks**

Potential risks/harms. Risks from this study include the loss of confidentiality in reporting potentially sensitive health information. Having a smartphone in their possession may also make participant targets for theft or exploitation. All smartphones will require an unlocking password and will be inaccessible without that password. Each participant and project personnel will be allocated a password to authenticate correspondence and prevent unauthorized parties from accessing information or providing false information. The health advice participants receive through the EMAI application may not be accurate, relevant, and may cause unintended harm. There is also risk of embarrassment, personal discomfort, and sensitivity to discussing and answering questions related to sexual behaviors and other sensitive subjects. Subjects may also become fatigued from interviews and meetings. The likelihood of these risks is small based upon previous RHSP studies and experiences.

Protection against risks. Recruitment and informed consent: Recruitment and informed consent: Participants provide written informed consent for RCCS, the parent study within which this study is nested. RCCS participants provide consent for recontact for other unspecified studies. Potential participants for this study will be approached by trained research staff. Staff will read, review, and discuss the study purpose, subject involvement, risks, potential benefits, and new consent forms specific to this study with potential subjects. All discussions and written material will be in the native language of the region (Luganda). All translations will be certified by a nationally recognized organization (Makerere University). If the participant agrees and is able to express a summary and understanding of the study, they will sign the consent form and be given a copy. Signed consent forms will be secured at the RHSP offices in Rakai.

Protection of confidentiality: *Research materials:* Materials will include the interviews, consent forms, and mobile phone logs of all activity conducted through the mHealth application. In order to preserve confidentiality, informed consent documents – which by definition include the participant's name - are retained in locked filing cabinets and secure store rooms, accessible only to senior investigators or designated staff. No personal identifiers will be attached to the questionnaire or samples. Electronic data sets and clinical readouts are downloaded into password protected computers, and individual identifiers are stripped. Names linked to study IDs are stored in a separate, password protected electronic file, on a computer and server accessible only to authorized senior personnel. All RHSP research staff have received extensive human subjects/research ethics training, including the need for complete confidentiality. Participant entered EMAI data will be encrypted at all times, be it on the phone, with data transfer, or sitting on the server. The application and server access will be password protected. If a phone is lost or stolen, we will have the ability to remotely wipe the phone of all data and render it inoperable. All interviews will be conducted in private or, if appropriate, as a focus group. If a breach of confidentiality occurs, we will report to the appropriate IRBs according to NIH guidelines. The principal investigator will discuss any breaches of confidentiality and intervene as necessary to decrease the risk of future breaches.

Avoidance of social harm: We will counsel participants on the need to be discrete with their smartphones to avoid risk of being targeted for theft or exploitation. We will attempt to use locally available smartphones to minimize the novelty of participants using these devices. We will teach participants to immediately contact study staff if they have any negative experiences and they will be able to return the phones and quit the study at any time.

Protection from discomfort, embarrassment, and fatigue: To mitigate these concerns, the following procedures will be implemented: (i) Training of research staff will emphasize caring, non-judgmental, and non-threatening approaches to the questioning, counseling, and teaching of patients; (ii) Research staff will be trained to stop their tasks if the patient appears to be in distress and to contact higher-trained staff if any distress is noted; (iii) Participants will be informed that they have the right to withdraw from the study at any time without any adverse consequences.

Protection from unintended consequences: Participants may encounter a variety of complex and unanticipated situations in the field once they begin this pilot study. Study team members will be available at all times via mobile phone for any situations which may arise. Participants may be confused by health messaging or have unintended harm from health messaging this is incorrect or inappropriate. RHSP health facilities are and will continue to be available to all participants free of charge and participants will be instructed to seek the advice of a health provider should there be any confusion or health concern. Overall, the EMAI is more likely to provide benefit rather than unintended harm. Additionally, our current and published experiences with EMAI have not found significant instances of harm.

mHealth-Specific Concerns: Special mention is made here regarding the ethical issues around EMAI, particularly the "tracking" aspects and use of geolocated data which will be linked to sensitive behaviors. In addition to full informed consent, the application, phones, and server will use rigorous security methods (passwords, encryption, remote data wiping, user controls, etc.) to help protect subjects. All geolocation data will be presented in anonymized and de-identified fashions (e.g. blurring specific locations to prevent identification). In the secondary objectives, we will specifically ask participants for their perspectives on the ethical issues of EMAI.

Data and Safety Monitoring Plan

The risks of this study are minimal compared to trials involving biomedical interventions or procedures. We have developed a data and safety monitoring plan in accordance with this lower level of risk. Primary responsibility for data and safety monitoring will be assumed by the principal investigator (Dr. Chang).

Data risks and monitoring: The primary data risks are inadequate quality of data collection, unacceptable intervention acceptance rates, data security, and unintended harm of health messaging. To address these risks, the principal investigator will review data quality and intervention acceptance rates on a quarterly basis. If risks are realized, investigators will consider stopping the study and/or taking appropriate steps to improve study quality and ensure adequate power. Electronic data security will be maintained with password protected computers accessible only to relevant study staff. Transcribed data will be secured in designated locked facilities at RHSP. Any data risk will be reported in a timely fashion to the appropriate IRBs. Any possible harm reported by participants will be immediately addressed by the PI and study team and reported to appropriate IRBs.

Safety risks and monitoring: The primary risks in this study are social harms related to loss of confidentiality and embarrassment and sensitivity related to discussions about sensitive topics such as health and sexual behaviors. To minimize these risks, we will perform informed consent and utilize a strong training curriculum that emphasizes strict confidentiality and protecting against inadvertent

disclosure. Additionally, we will ensure that training emphasizes caring, non-judgmental, and non-threatening approaches to the questioning, counseling, and teaching of participants. We will train study-related personnel to stop their tasks if the participant appears to be in distress and to contact higher-trained staff if distress is noted. Any breaches of confidentially will be considered an adverse event and immediately reported to the principal investigator. Should any adverse event occur which may be related to study activities, an adverse event form will be completed by study staff and the event immediately reported to the principal investigator.

Adverse Events: Adverse events will be routinely monitored, collected, and reported by study staff and the principal investigator according to NIH guidelines. Staff will be trained to complete an adverse event form which will be filed and electronically sent to the principal investigator for review. The principal investigator will ensure that study staff are appropriately trained on adverse event reporting. Adverse events will be reported promptly to the appropriate IRBs.

9. Benefits

Probable benefits of the proposed research to participants and for society. Subjects in this study may benefit from positive health messaging which may modify their health behaviors in a beneficial direction. If this intervention is demonstrated to be beneficial, there are potential benefits to the larger society and public health arena. For these reasons, the potential benefits outweigh the relatively minimal risks of this study.

Importance of knowledge to be gained. This is the first study to our knowledge of EMAI in sub-Saharan Africa. If this novel data collection and intervention method is proven to be feasible and acceptable, it could serve as an innovative platform for the future delivery of real-time, tailored, geolocated mHealth interventions for any disease at any stage in the continuum of health care and prevention, a potentially profound method for improving health.

10. Payment and Remuneration

We will provide incentives, given after completing each questionnaire, to optimize response rates. Overall, compensation will be approximately $30 over 90 days, given in three separate increments after Days 30, 60, and 90. If a participant fails to complete at least 80% of the prompts, they will forfeit their compensation.

Participants are also provided compensation for their participation in RHSP/RCCS activities and will receive this compensation per current RHSP/RCCS guidelines (currently a lump sum of about $4 for completing one survey round).

11. **Costs**-Not applicable.

References

1. Yonazi E, Kelly T, Halewood N, Blackman C. The Transformational Use of Information and Communication Technologies in Africa: The World Bank and the African Development Bank, with the support of the African Union, 2012.
2. Beratarrechea A, Lee AG, Willner JM, Jahangir E, Ciapponi A, Rubinstein A. The impact of mobile health interventions on chronic disease outcomes in developing countries: a systematic review. *Telemedicine journal and e-health : the official journal of the American Telemedicine Association* 2014; **20**(1): 75-82.
3. Déglise C, Suggs SL, Odermatt P. Short Message Service (SMS) Applications for Disease Prevention in Developing Countries. *J Med Internet Res* 2012; **14**(1): e3.
4. Alsallaq RA, Baeten JM, Celum CL, et al. Understanding the potential impact of a combination HIV prevention intervention in a hyper-endemic community. *PloS one* 2013; **8**(1): e54575.
5. Stone AA, Shiffman S, Atienza AA, Nebeling L. The Science of Real-Time Data Capture. New York: Oxford University Press, Inc.; 2007.
6. Linas BS, Latkin C, Westergaard RP, et al. Capturing illicit drug use where and when it happens: an ecological momentary assessment of the social, physical and activity environment of using versus craving illicit drugs. *Addiction* 2014.
7. Kirk GD, Linas BS, Westergaard RP, et al. The exposure assessment in current time study: implementation, feasibility, and acceptability of real-time data collection in a community cohort of illicit drug users. *AIDS research and treatment* 2013; **2013**: 594671.
8. Yang C. Ecological momentary assessment of alcohol use and sexual behaviors among men who have sex with men. mHealth Summit; December 8, 2014.
9. Labrique A, Vasudevan L, Chang LW, Mehl G. H_pe for mHealth: more "y" or "o" on the horizon? *International journal of medical informatics* 2013; **82**(5): 467-9.
10. Bollinger RC, McKenzie-White J, Gupta A. Building a global health education network for clinical care and research. The benefits and challenges of distance learning tools. Lessons learned from the Hopkins Center for Clinical Global Health Education. *Infect Dis Clin North Am* 2011; **25**(2): 385-98.
11. Togun T, Peterson I, Jaffar S, et al. Pre-treatment mortality and loss-to-follow-up in HIV-1, HIV-2 and HIV-1/HIV-2 dually infected patients eligible for antiretroviral therapy in The Gambia, West Africa. *AIDS research and therapy* 2011; **8**(1): 24.
12. Uganda PEPFAR Distance Learning Initiative. Available at http://ugandaghen.ccghe.net/.
